# High-Grade B-Cell Neoplasm with Surface Light Chain Restriction and Tdt Coexpression Evolved in a* MYC*-Rearranged Diffuse Large B-Cell Lymphoma: A Dilemma in Classification

**DOI:** 10.1155/2017/6891957

**Published:** 2017-08-13

**Authors:** Dina Sameh Soliman, Ahmad Al-Sabbagh, Feryal Ibrahim, Ruba Y. Taha, Zafar Nawaz, Sarah Elkourashy, Abdulrazzaq Haider, Susanna Akiki, Mohamed Yassin

**Affiliations:** ^1^Department of Laboratory Medicine and Pathology, National Center for Cancer Care and Research, Hamad Medical Corporation, Doha, Qatar; ^2^Department of Clinical Pathology, National Cancer Institute, Cairo University, Cairo, Egypt; ^3^Department of Hematology and Medical Oncology, National Center for Cancer Care and Research, Hamad Medical Corporation, Doha, Qatar

## Abstract

According to World Health Organization (WHO) classification (2008), B-cell neoplasms are classified into precursor B-cell or a mature B-cell phenotype and this classification was also kept in the latest WHO revision (2016). We are reporting a male patient in his fifties, with tonsillar swelling diagnosed as diffuse large B-cell lymphoma (DLBCL), germinal center. He received 6 cycles of RCHOP and showed complete metabolic response. Two months later, he presented with severe CNS symptoms. Flow cytometry on bone marrow (BM) showed infiltration by CD10-positive Kappa-restricted B-cells with loss of CD20 and CD19, and downregulation of CD79b. Moreover, the malignant population showed Tdt expression. BM Cytogenetics revealed t(8;14)(q24;q32) within a complex karyotype. Retrospectively, MYC and Tdt immunostains performed on original diagnostic tissue and came negative for Tdt and positive for MYC. It has been rarely reported that mature B-cell neoplasms present with features of immaturity; however the significance of Tdt acquisition during disease course was not addressed before. What is unique in this case is that the emerging disease has acquired an immaturity marker while retaining some features of the original mature clone. No definitive WHO category would adopt high-grade neoplasms that exhibit significant overlapping features between mature and immature phenotypes.

## 1. Introduction

The accurate classification of lymphoid neoplasms is vital for determining subsequent therapy and requires a multiparametric approach blending clinical, morphologic, immunophenotypic, and cytogenetic/molecular data to formulate a final diagnosis. Diffuse large B-cell lymphoma (DLBCL) is a diverse disease that had been subdivided into biologically heterogeneous subgroups based on morphological, molecular, and immunophenotypic diversity.

In the diagnostic evaluation of B-cell neoplasms, flow cytometric and immunohistochemical immunophenotyping have a critical role in the differentiation of a precursor B-cell phenotype from a mature B-cell phenotype [1]. The most common types of mature B-cell neoplasms are DLBCL and follicular lymphoma (FL) (excluding Hodgkin's lymphoma and plasma cell myeloma) [2]. Tdt and CD34 are considered as surrogate immaturity markers while surface light chain restriction generally indicates a mature phenotype. Burkitt lymphoma (BL) is an aggressive B-cell neoplasm. Most of BL cases (90%) harbor the characteristic* MYC* translocation t(8;14)(q24;q32) which juxtaposes the* MYC *(previously known as* cMYC*) gene normally located at 8q24 with the* IG *heavy-chain locus, as well as the so-called variant translocations, t(2;8)(p12;q24) and t(8;22)(q24;q11), involving the* IG *light chain genes; these are considered genetic hallmarks of BL.* MYC* rearrangements can also be found in DLBCL [3] and even in precursor B-lymphoblastic leukaemia/lymphoma (B-ALL/LBL) [4]. B-ALL is a neoplasm of B-lymphoblasts that are characteristically negative for surface immunoglobulins and express immaturity markers and markers related to the degree of B-cell differentiation. Few cases of B-ALL/LBL with surface light chain restriction have been previously reported [5].

Herein, we report unique case of an aggressive* MYC*-rearranged high-grade B-cell neoplasm with monotypic surface light chain and Tdt coexpression along with loss of pan B-cell antigens (CD19, CD20) evolved in a patient with two-month history of diffuse large B-cell lymphoma, germinal center (GC) origin.

## 2. Case Report

**Figure 1:**
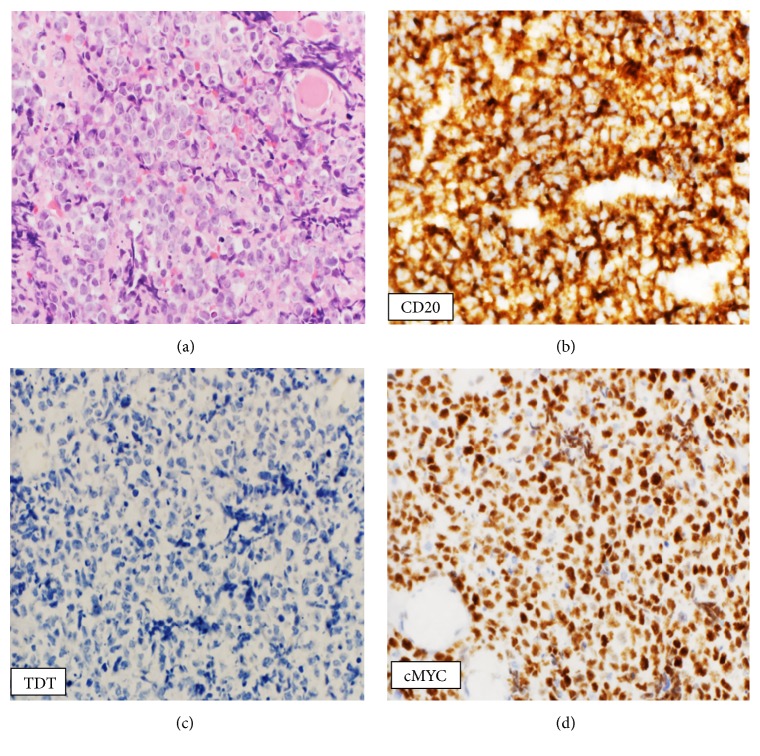
Tosillar tissue: H&E sections showing large to medium sized lymphoid cells with round to oval vesicular nuclei (a). The malignant cells are positive for CD20 immunostain (b). Immunohistochemistry (40x): Retrospectively performed on original diagnostic tonsillar tissue. Negative for TDT (c) and positive for MYC immunostains (d).

Our patient is a male in his fifties with type II diabetes, dyslipidemia, and chronic hepatitis C who presented in 2016 with a two-month history of odynophagia and neck swelling with no B-symptoms. On physical examination, a left para-tonsillar swelling was noted. Further, computerized tomography (CT) imaging showed a large soft tissue mass extending from the left tonsillar fossa and inferiorly to the left parapharyngeal wall. Magnetic resonance imaging (MRI) redemonstrated the pharyngeal mass but also bilateral cervical lymphadenopathy. The patient subsequently underwent a pan-endoscopy and excisional biopsy of the pharyngeal mass. Morphologic and immunohistochemical studies revealed a final diagnosis of DLBCL, germinal center (GC) subtype. Histopathological examination showed medium sized to large lymphoid cells with round to oval vesicular nuclei containing single to multiple nuclei (Figure 1(a)). The malignant lymphoid cells were positive for CD20 (Figure 1(b)), CD79a, PAX-5, BCL-2, BCL-6, and CD10.* cMYC* immunostain was not performed at time of diagnosis. Laboratory investigations including complete blood counts (CBC), electrolytes, and renal and liver function tests were unremarkable except for a thrombocytopenia of 102 × 10^3^/*μ*L (150–400) and slightly elevated Lactate dehydrogenase (LDH) (274.0 U/L; *n*: 135–225).

Bone marrow examination (BM) was performed as a part of staging work-up and showed small atypical CD20-positive lymphoid aggregates; however BM involvement could not be confidently concluded due to suboptimal specimen quality. CSF cytologies were negative for lymphomatous involvement. The patient was diagnosed as oropharyngeal DLBCL, unconfirmed stage I, International Prognostic Index (IPI) 1-2, and he subsequently received treatment with RCHOP for 21 days and CNS prophylaxis with intrathecal (IT) methotrexate. The patient completed 6 cycles of therapy, with uncomplicated course. Mid-treatment and end of treatment Positron Emission topography (PET/CT) showed complete metabolic response.

Two months later, the patient presented with subacute onset bilateral upper limb weakness, which progressed to dense right-sided upper limb weakness, complete left sided facial weakness, and blurred vision (bilateral 6th nerve palsies). He also had a decreased level of consciousness and intermittent tonic-clonic seizures, no headache, neck stiffness, or vomiting. CT and MRI scans of the brain were unremarkable.

CBC showed thrombocytopenia (94 × 10^3^/*μ*L); with mild leukopenia (3.6 × 10^3^/*μ*L; 4–10). LDH was elevated at 257.0.

PET/CT imaging was unremarkable except for mild subfrontal increase uptake (possibly leptomeningeal involvement).

**Figure 2:**
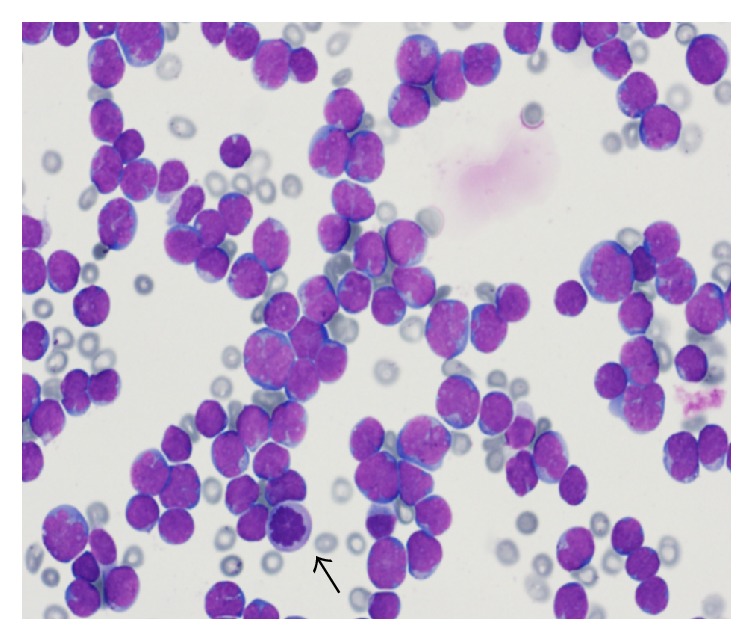
CSF cytospin preparation smear (50x) shows infiltration with many malignant lymphoid cells, some of which are larger with irregular nuclear contours, less clumped nuclear chromatin, and prominent nucleoli with few mitotic figures (black arrow) (Wright stain, ×500).

A lumbar puncture showed marked lymphocytic pleocytosis (WBC: 651/ul; *n*: 0–5) and CSF cytology showed many malignant lymphoid cells, compatible with relapsed lymphoma (Figure 2).

**Figure 3:**
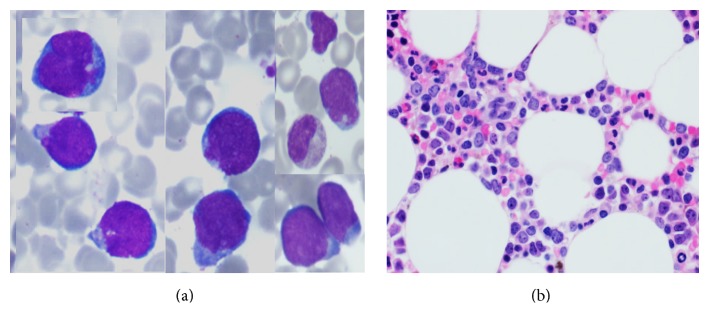
BM aspirate smear shows numerous abnormal medium to large-sized lymphoid cells. The cells showed slightly irregular nuclear contours, dispersed nuclear chromatin, and basophilic cytoplasm (Wright stain, 1,000x) (a). BMB biopsy (H&E 50x): interstitial infiltration with malignant lymphoid cells (b).

**Figure 4:**
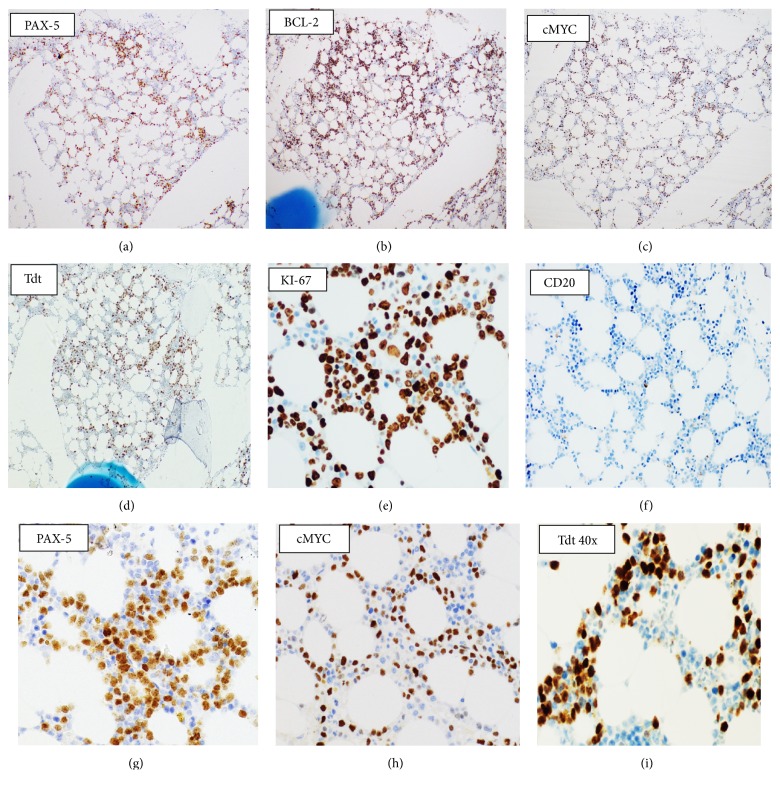
Immunohistochemistry performed on bone marrow biopsy (first relapse). The abnormal lymphoid cells are positive for PAX-5, BCL-2,* cMYC*, and TDT (a–d). KI-67 shows high proliferative index (e). The neoplastic cells are negative for CD20 (f). PAX-5 50x (g), cMYC 40x, and Tdt 40x (i).

**Figure 5:**
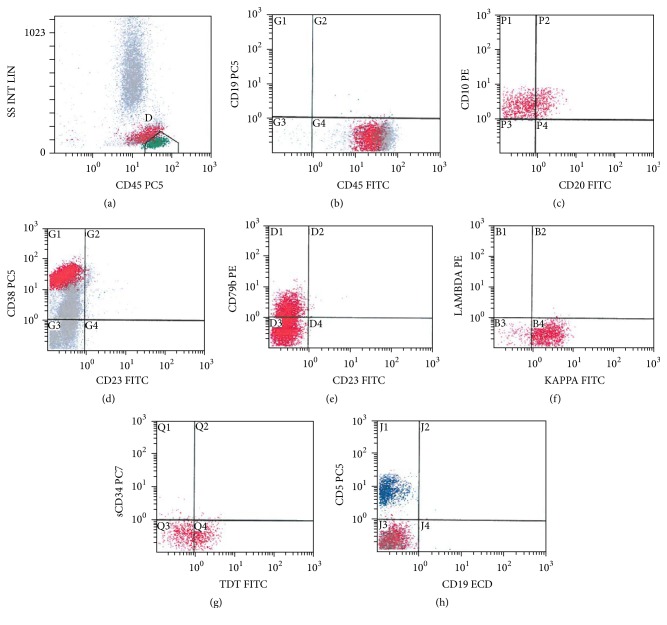
Flow cytometry on bone marrow aspirate: a population of monotypic B-cells, expressing CD45, CD10, CD38, and surface kappa light chain restriction. The monotypic B-cells are negative for CD 5 with downregulation of pan B markers (partial CD79 (dim), loss of CD19 and CD20).

Restaging BM showed infiltration by abnormal medium to large-sized lymphoid cells (~40%) with slightly irregular nuclear contours, dispersed nuclear chromatin, few nucleoli, and basophilic cytoplasm (Figure 3(a)). Few cells also showed prominent cytoplasmic vacuolation. The BM biopsy was hypocellular (20–30%) with interstitial infiltration by many malignant lymphoid cells (Figure 3(b)). By immunohistochemistry (IHC), the lymphoid cells were positive for PAX-5, BCL-2 (more than 50%),* cMYC, and* Tdt immunostains (Figures 4(a)–4(d)) with high mitotic index reflected by strong KI-67 positivity (Figure 4(e)). The neoplastic cells were negative for CD20 (Figure 4(f)), CD5, BCL6, CD23, MUM-1, and Cyclin D1. Flow cytometry (FCM) of the BM aspirate (Figure 5) revealed a population of kappa-restricted monotypic B-cells (~15%), expressing CD45, CD10, and CD38 (bright) and showed surface kappa light chain restriction. The monotypic B-cells are negative for CD5 and showed downregulation of pan B markers (partial expression of CD79 (dim), loss of CD19 and CD20). Moreover, the malignant population showed partial dim expression of Tdt (Figure 5(g)). FCM on CSF showed infiltration with malignant cells with the same phenotype.

**Figure 6:**
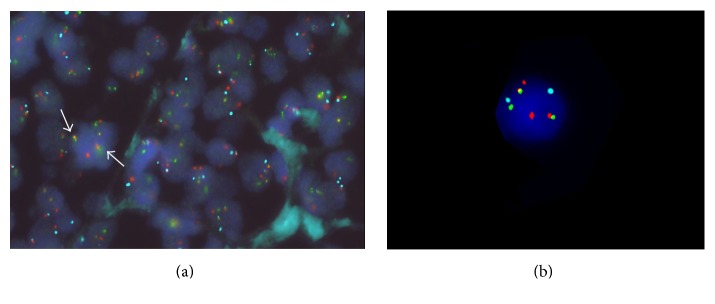
CEP8/MYC/IGH probes on tonsils (a) and bone marrow biopsy (b). The FISH analysis was performed using probes from Vysis; MYC-Orange spectrum, CEP8-Aqua spectrum, and IGH-Green spectrum. White arrows indicate the fusion signal a result of MYC/IGH translocation.

Conventional cytogenetics studies revealed a complex karyotype: (48,XY,+X,t(8;14)(q24;q32),+12,13,der(14)t(1;14)(q21;p11.2),+der(?)t(1;?)(q21;?)[8]/50,idem,+der(?)t(1;?),+mar[2]/46, XY[22]) and subsequent FISH analysis confirmed the* MYC*/*IGH*, t(8;14) rearrangement in 41% of cells analyzed (Figure 6(b)). There was no evidence of* BCL*-6 or* BCL*-2 gene rearrangement.

Retrospectively, MYC and Tdt immunostains performed on original diagnostic tissue and came positive for MYC and confirmed negativity for Tdt (Figures 1(c) and 1(d)).

Screening of the original diagnostic tonsillar tissue for double hit gene rearrangement* IGH/BCL2, MYC/IGH* by FISH analysis was also performed at this stage and revealed positivity for MYC/IGH (Figure 6(a)) and negativity for BCL-2 gene rearrangement. Unfortunately, additional molecular studies were not available in our centre.

A final diagnosis of* MYC*-rearranged high-grade B-cell neoplasm stage IV (leptomeningeal and bone marrow involvement), IPI 4, with Tdt expression, likely representing early relapse/transformation of previous* MYC*-rearranged DLBCL was agreed upon at a multidisciplinary meeting.

The patient received triple intrathecal (Methotrexate/Cytarabine/Hydrocortisone) chemotherapy twice weekly till clearance of CSF from malignant cells. Concurrently, he started on high-dose methotrexate therapy alternating with high-dose Cytarabine (total of four cycles). Mid-treatment BM examination showed no evidence of disease. The therapy was complicated with decompensated liver failure manifested with hyperbilirubinemia, encephalopathy, and multiple sepsis with* Pseudomonas aeruginosa* and accordingly the patient was not a candidate for consolidation with high-dose therapy and SCT.

**Figure 7:**
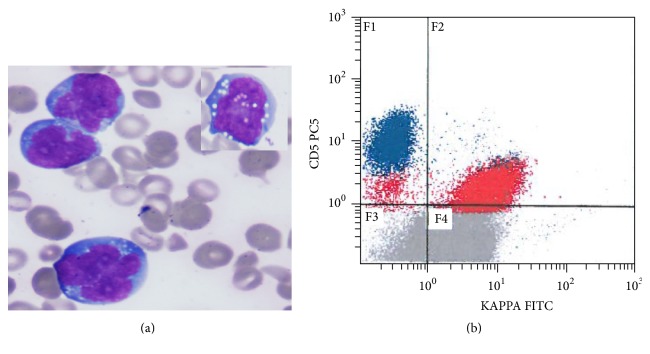
Peripheral smear at time of second relapse: Neoplastic cells show more pronounced nuclear irregularities with variable cytoplasmic vacuolation (a). Wright stain, 1,000x magnification. Flow cytometry on peripheral blood showed malignant cells with CD5 acquisition (b).

The patient was maintained throughout the treatment on extensive physiotherapy program. After recovery from last cycle of chemotherapy, he started to walk independently. Unfortunately, the patient relapsed again within few weeks where circulating malignant cells ~10% were detected in peripheral smear (Figure 7(a)), for which flow cytometry was performed and revealed a population of monotypic B-cells ~10% expressing CD45, CD10, CD20, and CD38, with kappa light chain restriction, loss of CD19, and acquisition of CD5 expression (Figure 7(b)). Shortly after, the patient passed away and this was four months after his first relapse.

## 3. Discussion

According to World Health Organization (WHO) classification system for Hematopoietic and Lymphoid neoplasms (2008) [6], neoplasms of the B-lymphoid cell lineage can be broadly classified into those having a precursor B-cell or a mature B-cell phenotype and this is also kept in the latest WHO 2016 update in which Tdt expression was considered exclusive for precursor B-cell neoplasms [7]. MYC gene is rearranged in 5% to 15% of DLBCL, NOS, and is frequently associated with BCL2 or, to a lesser extent, BCL6 translocation, in the so-called “double hit” (DH) or “triple-hit” lymphomas that are included in the updated revision of WHO classification within the new category of high-grade B-cell lymphoma (HGBL), with rearrangements of MYC and BCL2 and/or BCL6 [7, 8]. MYC protein expression is detected in a much higher proportion of DLBCL (30%–50%) and is associated with concomitant expression of BCL2 in 20% to 35% of cases [9]. Most of these tumors do not carry MYC/BCL2 chromosomal rearrangements and have been labelled as “double-expressor lymphoma (DE).” There is some controversy about the prognosis of DE lymphomas, however; several studies showed that this category has an inferior outcome compared to other DLBCL, NOS, but they are not as aggressive as the HGBL with rearrangements of MYC and BCL2 and/or BCL6 [10, 11].

Back to our case, which demonstrated quite unusual morphologic, immunophenotypic, and cytogenetics combination, it was rather challenging to classify the disease according to WHO, to ascertain the cell of origin or to link it to original disease (at presentation).

Given the prior history of recently diagnosed DLBCL (GC), the most relevant diagnosis was relapsing DLBCL. However, in view of the aggressive presentation with systemic involvement and the marked immunophenotypic aberrancies (including Tdt expression), a simple diagnosis of relapsed DLBCL could not be committed.

An alternative scenario was to consider the latter presentation as a different emerging immature clone unrelated to original disease. This proposition was supported by the purely extranodal involvement (BM and CSF), aggressive behavior, and immunophenotypic differences especially the acquisition of Tdt, the hallmark of cell immaturity. For the latter reason, B-lymphoblastic leukaemia/lymphoma (B-ALL) was considered in the differential diagnosis.

Despite the unequivocal expression of Tdt (by IHC and FCM) and the well documented expression of surface immunoglobulin in cases of B-lymphoblastic lymphoma [3], in addition to the fact that classic MYC translocation can be detected in about 7% of B-ALL/LBL cases [12] and its presence by no means excludes a diagnosis of B-ALL/LBL, however, there was a more convincing evidence that we are dealing with a mature B-cell neoplasm that acquired Tdt expression as an immunophenotypic drift or as a manifestation of higher grade transformation/clonal evolution rather than a novel immature clone. This was supported by lack of any additional morphologic or immunophenotypic features that are supportive of immaturity; as the malignant cells were not really having blastoid morphology, with bright CD45 expression, negative CD34, and positive surface immunoglobulin with kappa light chain restriction. In addition, loss of CD20 could not be considered as supportive evidence of immaturity as this could be simply related to Rituximab treatment rather than de novo, especially that the cells had regained CD20 expression in second relapse. All the aforementioned features made the diagnosis of denovo B-ALL less likely.

Regain of CD20 within relatively short time from Rituximab therapy and the acquisition of CD5 later in the course of the disease would suggest a malignant clone with high genomic instability evidenced by the complex karyotype detected.

Putting together the morphologic, immunophenotypic, and cytogenetic findings as well as the prior history of recently diagnosed DLBCL with triple BCL-2, BCL-6, and cMYC protein expression (on the original diagnostic tissue), the diagnostic category of “B-cell lymphoma unclassifiable, with features intermediate between DLBCL and BL (BCLU)” (WHO 2008) was proposed.

According to the revised WHO (2016) [7], it was again difficult to confidently categorize this disease; however the three most relevant categories are as follows:

 
*Diffuse large B-cell lymphoma, NOS*, with coexpression of MYC and BCL2 protein.
 
*Or high-grade B-cell lymphoma, with MYC and BCL2 and/or BCL6 rearrangements*^*∗*^: includes all “double-/triple-hit” lymphomas other than FL or lymphoblastic lymphomas.
 
*Or high-grade B-cell lymphoma, NOS*^*∗*^: for cases intermediate between DLBCL and BL, but lack a MYC and BCL2 and/or BCL6 rearrangement.

In the case reported here, the first presentation (but not at relapse) would probably fit into the first category with coexpression of MYC and BCL2 protein (double-expressor lymphoma). The absence of solid evidence of BCL2/BCL-6 gene rearrangements by FISH or standard cytogenetics together with detection of classic MYC translocation and coexpression of surface immunoglobulin and Tdt: the latter presentation would not fit into any of the previously listed categories as all should be negative for Tdt.

Even though no definite final classification could be confidently made, we considered the case as high-grade B-cell neoplasm probably representing a clonal evolution/transformation/dedifferentiation from* MYC*-R DLBCL with acquired Tdt expression.

*MYC*-R DLBCL have a higher rate of CNS involvement at presentation and posttreatment [13, 14]. Furthermore, a recent study [15] demonstrated that coexpression of MYC and BCL2 by IHC in DLBCL predicted risk of CNS relapse independent of other established variables. However, thus far there are no unique clinical or pathologic features that can identify* MYC*-R lymphomas in practice. However, a recent study [16] showed that bright CD38 expression and dim CD45 expression are unique to* MYC*-R and not non-*MYC*-R DLBCL.

The loss of pan-B-cell markers in our case was also rather peculiar. The vast majority of de novo cases of mature B-cell lymphoma express CD20 and/or CD19 although any one of these pan-B-cell markers may be rarely lost [17, 18]. The most common clinical scenario where CD20 loss is seen in mature B-cell lymphomas is in the relapse/recurrence setting post-Rituximab therapy months or even years after therapy [19]. While CD20 loss can possibly be explained by Rituximab effect, concurrent absence of CD19 and CD20 and downregulation of CD79 are extremely rare in mature B-cell neoplasms neither at initial diagnosis nor at relapse. CD19-cre/Pax5fl/- mice, which lose Pax5 in CD19 positive cells, die within 8 months of birth from aggressive lymphomas that express B220 and Ig *μ* but lack CD19 and other B-cell markers [20].

Rare cases of DHL presenting with Tdt expression have been reported, most with the morphology and immunophenotype of B-LBL, arising from or presenting simultaneously with FL [21–23]. Loghavi et al., 2015 [24], have reported five cases of Tdt-positive B-cell neoplasms with* BCL2 *rearrangements and* MYC *rearrangements; four of these had high-grade blastoid neoplasms at presentation while the fifth case was transformed from preceding follicular lymphoma. All these reported tumors were positive for CD10, CD19, and Tdt and negative or only dimly positive for CD20 and importantly negative for surface immunoglobulins.

Blastic transformation of FL to BL is a rare event but has been documented for more than 25 years [25]. DHL arising from or presenting concurrently with FL has also been rarely reported [14, 21, 24–26]. On the other hand, transformation of DLBCL (GC) into B-LBL is a very rare event. The mechanism of transformation of FL is not well understood and different models had been proposed.

A mechanism of “dedifferentiation” of lymphoma cells into more immature stages as a result of secondary genetic events has been suggested in our case. Although no molecular studies were performed as this test is not available in our institution, the immunophenotypic and genetic similarities between the original neoplasm and the second presentation (including mature phenotype, CD10 positivity, BCL-2 expression, and* cMYC* gene rearrangement) have suggested that the two neoplasms probably had originated from a common B-cell clone or the second presentation has emerged from clonal evolution of its former DLBCL and “dedifferentiated” into more immature stage. Another possibility is that there was an additional minor undetected clone at time of presentation.

It has been rarely reported that MYC-rearranged B-lymphomas can present with features suggestive of immaturity, including Tdt expression. However, the significance of Tdt acquisition during disease course/relapse was not clearly addressed before. What is unique in this case is that the emerging disease has acquired an immaturity marker (Tdt) while keeping some immunophenotypic and cytogenetics features of the original mature clone.

## 4. Conclusion

In conclusion, in this case, the changes in the biological characteristics of tumor cells postchemotherapy and acquisition of immunophenotypic aberrancies including Tdt expression had led to early relapse with aggressive clinical presentation and fatal outcome. The importance of early recognition of MYC expression (which might have modified the treatment protocol) is questionable. An extensive literature review did not identify a similar case of a B-cell neoplasm with such unusual immunophenotypic aberrancies (particularly Tdt expression) that likely clonally evolved from MYC-R DLBCL. Reporting this case with such peculiar immunophenotypic features would probably spotlight cases of Tdt-positive mature B-cell neoplasms. Although these cases were rarely reported, there is no definitive WHO category that can adopt such high-grade neoplasms with significant overlapping features between mature and immature phenotypes.

As Tdt is a hall mark of immaturity and considered as a differentiating marker between mature and immature B-cell neoplasms (WHO 2016), we recommend including Tdt in the routine immunophenotyping panel in cases of aggressive mature B-cell neoplasms particularly DH and DE DLBCL to recognize those aggressive neoplasms which lack solid clinicopathological characterization in the literature, to draw more attention to the effect of Tdt expression on treatment response and disease outcome. It is also of interest to identify the probable molecular mechanisms implemented in disease pathogenesis and exploring the role of “dedifferentiation” which has not been previously discussed in context of DLBCL transformation.
